# Supplementary material for: Integrative Kinase Activity Profiling and Phosphoproteomics of rd10 Mouse Retina during cGMP-Dependent Retinal Degeneration
Source: Int J Mol Sci. 2024 Mar 19;25(6):3446. doi: 10.3390/ijms25063446 (PMC10970885; doi:10.3390/ijms25063446)
Supplement: Supplementary file 1 [file ijms-25-03446-s001.zip › ijms-2890265-supplementary.pdf]

Supplementary Data

Supplementary Table S1: Kinase analysis from MS-based Phosphoproteomic results for *rd10* vs. *rd10* CN03

	Kinase Name	Kinase Uniprot ID	Log(fold change)	-log10(p-val)
1	MAPK14	Q16539	-1.68	3.91
2	AKT1	P31749	0.76	3.41
3	RPS6KA1	Q15418	0.79	3.00
4	AURKB	Q96GD4	0.73	2.96
5	ZAP70	P43403	-1.43	2.91
6	PRKCA	P17252	0.59	2.55
7	PNCK	Q6P2M8	0.69	2.54
8	CDK2	P24941	1.11	2.45
9	FYN	P06241	1.01	2.18
10	ATR	Q13535	0.90	2.10
11	GSK3B	P49841	0.57	2.04
12	CSNK1A1	P48729	0.57	2.04
13	PLK3	Q9H4B4	0.57	2.04
14	PRKACA	P17612	0.95	1.91
15	PRKCG	P05129	-0.64	1.89
16	RPS6KA3	P51812	0.58	1.88
17	MKNK1	Q9BUB5	0.65	1.80
18	RPS6KB2	Q9UBS0	0.65	1.80
19	ATM	Q13315	0.95	1.77
20	GRK6	P43250	0.65	1.61
21	RPS6KB1	P23443	1.27	1.47
22	CAMK2A	Q9UQM7	-1.74	1.46
23	MAPKAPK2	P49137	-0.96	1.41
24	MAPK1	P28482	-0.98	1.34
25	MAPK3	P27361	-0.98	1.34
26	ABL1	P00519	-0.98	1.34
27	TRPM7	Q96QT4	0.64	1.33
28	CSNK2B	P67870	0.64	1.33

Supplementary Table S2: List of antibodies used in Western blotting

Target protein	Antibody	Molecular weight	Supplier	Catalogue no.	Dilution
HSP90	HSP 90a/b (F08)	90 kD	Santa cruz biotechnologies	sc-13119	1:500
pCREB	Phospho-CREB (Ser133; F.959.4)	43 kDa	ThermoFisher Scientific	MA5-11192	1:1000
pCaMK2	Phospho-CaMKII beta/gamma/delta (Thr287)	54/60 kDa	ThermoFisher Scientific	PA5-37833	1:1000
pCaMK4	Anti-phospho-CaMK4 (pThr196/200)	51 kDa	Sigma Aldrich	SAB4504122	1:500
secondary antibody: HSP90	Anti-mouse-HRP	-	Santa cruz biotechnologies	SC-2005	1:50000
secondary antibody: pCREB, pCaMK2 and pCaMK4	Anti-Rabbit-HRP	-	Cell Signaling Technology	7074S	1:50000
